# Genetic Variants Relate to Fasting Plasma Glucose, 2-Hour Postprandial Glucose, Glycosylated Hemoglobin, and BMI in Prediabetes

**DOI:** 10.3389/fendo.2022.778069

**Published:** 2022-03-01

**Authors:** Leweihua Lin, Tuanyu Fang, Lu Lin, Qianying Ou, Huachuan Zhang, Kaining Chen, Huibiao Quan

**Affiliations:** Department of Endocrinology, Hainan General Hospital，Hainan Affiliated Hospital of Hainan Medical University, Haikou, China

**Keywords:** genome-wide association study, fasting plasma glucose, 2-hour postprandial glucose, glycosylated hemoglobin, prediabetes

## Abstract

Diabetes mellitus (DM) is a chronic disease that seriously threatens human health. Prediabetes is a stage in the progression of DM. The level of clinical indicators including fasting plasma glucose (FPG), 2-h postprandial glucose (2hPG), and glycosylated hemoglobin (HbA1C) are the diagnostic markers of diabetes. In this genome-wide association study (GWAS), we aimed to investigate the association of genetic variants with these phenotypes in Hainan prediabetes. In this study, we recruited 451 prediabetes patients from the residents aged ≥18 years who participated in the National Diabetes Prevalence Survey of the Chinese Medical Association in 2017. The GWAS of FPG, 2hPG, HbA1C, and body mass index (BMI) in prediabetes was analyzed with a linear model using an additive genetic model with adjustment for age and sex. We identified that rs13052524 in *MRPS6* and rs62212118 in *SLC5A3* were associated with 2hPG in Hainan prediabetes (*p* = 4.35 × 10^-6^, *p* = 4.05 × 10^-6^, respectively). Another six variants in the four genes (*LINC01648*, *MATN1*, *CRAT37*, and *SLCO3A1*) were related to HbA1C. Moreover, rs11142842, rs1891298, rs1891299, and rs11142843 in *TRPM3/TMEM2* and rs78432036 in *MLYCD/OSGIN1* were correlated to BMI (all *p* < 5 × 10^-6^). This study is the first to determine the genome-wide association of FPG, 2hPG, and HbA1C, which emphasizes the importance of in-depth understanding of the phenotypes of high-value susceptibility gene markers in the diagnosis of prediabetes.

## Introduction

Diabetes mellitus (DM) is an endocrine metabolic disease caused by many pathogenic factors (genetic and environmental factors included) marked by elevated blood glucose, becoming a global public health problem (1, 2). According to statistics, there are about 100 million DM over the age of 20 in China, which is expected to increase to 143 million by 2035 (3). Type 2 DM (T2D) is the main type of DM. Prediabetes is a special state between health and diabetes, in which blood glucose is elevated without meeting the diagnostic criteria for DM. Impaired glucose tolerance (IGT) and impaired fasting glucose (IFG) are high risk factors for developing prediabetes and are defined as the level of fasting plasma glucose (FPG), 2-h postprandial glucose (2hPG), and glycosylated hemoglobin (HbA1C) in diabetes (4). Moreover, people with prediabetes are more likely to develop T2D than the general population (5).

Genome-wide association study (GWAS), a common genetic analysis tool, provides insights into the molecular status of complex diseases such as T2D and supports risk prediction (6). For example, Choi et al. (7) used the GWAS to detect the association of single-nucleotide polymorphisms (SNPs) with microalbuminuria in patients with prediabetes, and they found two susceptibility loci related to prediabetes. Additionally, Soranzo et al. (8) used GWAS to identify 10 genetic loci associated with HbA1C at genome-wide levels of significance in European non-diabetic adults. Ge et al. (9) reported that 9 genetic loci were significantly related to an increased FPG level in T2D. Another study also showed that multiple GWAS variants were associated with FPG in African-American non-diabetes (10). Epidemic obesity is the most important risk factor for prediabetes (11). Taken together, we speculate that genetic predisposition to prediabetes might be associated with some risk factors such as FPG, 2hPG, and HbA1C. However, the association between FPG, 2hPG, HbA1C, and body mass index (BMI) and genetic variants has not been studied in Hainan prediabetes.

In this GWAS analysis, we recruited 451 prediabetes patients in Hainan province to investigate the association of genetic susceptibility loci with the phenotypes including FPG, 2hPG, HbA1C, and BMI. Our study will provide a new perspective for the prevention and diagnosis of diabetes in Hainan province.

## Materials and Methods

### Study Population

In this study, we recruited 451 prediabetes patients from the residents aged ≥18 years who participated in the National Diabetes Prevalence Survey of the Chinese Medical Association in 2017. The diagnostic criteria of prediabetes was 5.7% ≤ HbA1C < 6.4% or 140 mg/dl (7.8 mmol/L) ≤ 2hPG < 200 mg/dl (11.1 mmol/L) or 100 mg/dl (5.6 mmol/L) ≤ FPG < 126 mg/dl (7.0 mmol/L) (12). All the participants were informed of the purpose of the study and signed the written informed consent forms. Our study was approved by the Ethics Committee of Hainan Affiliated Hospital of Hainan Medical University [Med-Eth-Re (2019) 18], and all experiments were carried out in accordance with the standard protocol of Helsinki’s Declaration of 1964 and its later amendments.

A 75-g oral glucose tolerance test (OGTT) was performed to test the level of 2hPG in accordance with the protocol of a published article (13). The FPG level was tested using an automatic biochemical analyzer. The concentration of HbA1C was determined by high-performance liquid chromatography (HPLC).

### Genomic DNA Extraction and Genotyping

Genomic DNA was extracted from a whole-blood sample using a whole-blood genomic DNA purification kit (Xi’an GoldMag Nanobiotech Co., Ltd., Xi’an, Shaanxi, China). Genotyping was detected by Axiom™ Precision Medicine Diversity Array (PMDA; Thermo Fisher Scientific Technology Co., Ltd., Shanghai, China). Affymetrix Gene Titan was performed to genotype calling, and data were analyzed by Axiom Analysis Suite 6.0 software.

### Imputation and Quality Control

A total of 108,825 SNPs were obtained after genotyping. Quality control (QC) procedure was applied for genotyping. All individuals through the QC could be applied in the final statistical analysis. A total of 108,825 SNPs were obtained after genotyping. Among them, 103,506 SNPs met the criteria of the QC with sample call rate >0.95, maker call rate >0.90, and Hardy–Weinberg equilibrium (HWE) >5e-06, which were used for the final GWAS analysis. Genotype clustering was conducted by Axiom Analysis Suite 6.0 software. In addition, the genotype data were imputed with 1000 Genomes Project phase 3 reference panel by IMPUTE2 software (14, 15) to 9,378,219 SNPs. SNPs with a minor allele frequency (MAF) <1%, info >0.3, Indel, copy number variation (CNV), duplication, non-biallelic variants, and loci from sex chromosome were removed for the QC procedure of imputation. After imputation, we finally obtained 1,752,717 SNPs.

### Statistical Analyses

This study was based on the 3σ principle to remove the extreme values of FPG, 2hPG, and HbA1C; and the levels of these indexes were normalized using rank-based inverse normal transformations by the R package RNOmni. The associations between SNPs and each phenotype including FPG, 2hPG, HbA1C, and BMI were evaluated by a linear model using an additive genetic model with adjustment for age and sex. The value of *p* < 5 × 10^-8^ indicated a statistical significance in this GWAS analysis, and *p* < 5 × 10^-6^ suggested a suggestively significant genome-wide association. Predictions of the possible function of candidate SNPs were performed using the HaploReg v4.1 online tool (https://pubs.broadinstitute.org/mammals/haploreg/haploreg.php). The distribution of FPG, 2hPG, HbA1C, and BMI in different genotypes was evaluated by one-way analysis of variance (ANOVA), and *p* < 0.05 was set to be statistically significant.

## Results

### Study Subjects

In this study, a total of 451 prediabetes patients including 216 men and 235 women from Hainan province were recruited to evaluate the association of FPG, 2hPG, HbA1C, and BMI with 1,752,717 genotyped SNPs using a genome-wide association analysis. The average age of participants was 51.78 ± 14.49 years. The characteristics of the study population were shown in **Table 1**.

**Table 1:** General characteristics of study participants.

Characteristics	N (451)
Age (years)	51.78 ± 14.49
Sex	
Men	216 (47.9%)
Women	235 (52.1%)
FPG (mmol/L)	5.875 ± 1.42
HbA1c (%)	5.965 ± 0.97
2hPG (mmol/L)	9.662 ± 3.47
BMI (kg/m^2^)	24.26 ± 3.10

FPG, fasting plasma glucose; HbA1c, glycosylated hemoglobin; 2hPG, 2-h postprandial glucose; BMI, body mass index.

### Association of Genetic Variants With Fasting Plasma Glucose, 2-h Postprandial Glucose, Glycosylated Hemoglobin, and Body Mass Index in Prediabetes

**Figure 1:**
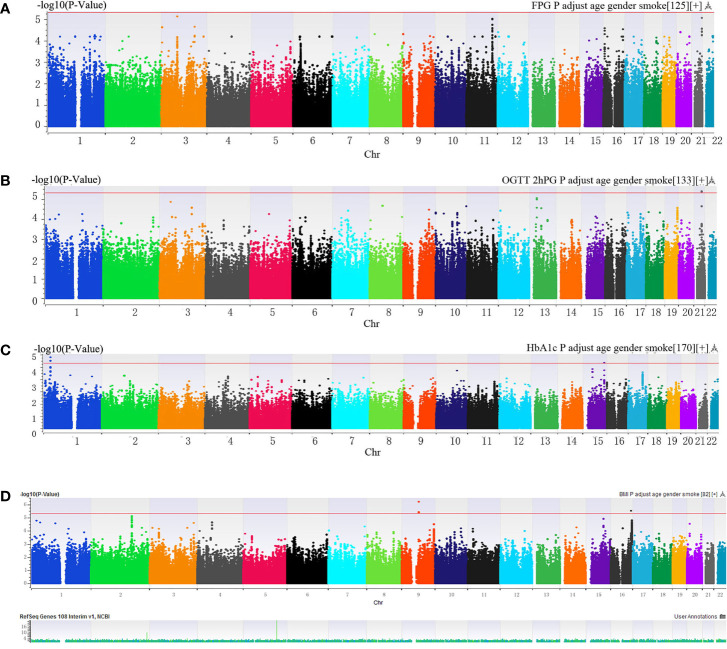
**(A–C)** Manhattan plot of genome-wide association analyses before imputation of **(A)** fasting plasma glucose (FPG), **(B)** 2-h postprandial glucose (2hPG), **(C)** glycosylated hemoglobin (HbA1C) and **(D)** BMI. The *x*-axis indicated human chromosomes, and the *y*-axis was the -log_10_ of the *p*-value. The red line indicates that the cutoff of the genome-wide significance is 5 × 10^-6^.

Manhattan plot (**Figure 1**) exhibited the chromosome location of suggestive loci for serum levels of 2hPG, HbA1C, and BMI. Genomic inflation factor (λ) was 1.010, 1.011, 1.015, and 1.013 for FPG, 2hPG, HbA1C, and BMI, respectively. A quantile-quantile (Q-Q) plot of the GWAS-analysis *p*-values shows that the test statistics follow null expectations and no small *p* values exceed expectations (**Figure S1**). After imputation, eight loci in different chromosomal regions with suggestive significance of *p* values <5 × 10^-6^ were shown in **Table 2**. Two SNPs (rs13052524, rs62212118) in the *MRPS6*/*SLC5A3* gene were associated with 2hPG (*p* = 4.35 × 10^-6^, *p* = 4.03 × 10^-6^; respectively). Furthermore, five SNPs, rs142013708, rs140071694, rs150306839, rs138084074, and rs142002616, in *LINC01648*/*MATN1* and rs11853125 in *CRAT37*/*SLCO3A1* were correlated to HbA1C (all *p* < 5 × 10^-6^). Moreover, rs11142842, rs1891298, rs1891299, and rs11142843 in *TRPM3/TMEM2* and rs78432036 in *MLYCD/OSGIN1* were correlated to BMI (all *p* < 5 × 10^-6^). The results of HaploRegv4.1 displayed that these SNPs might be associated with the regulation of promoter and/or enhancer histones, DNAse, changed motifs, and selected eQTL hits (**Table 2**).

**Table 2:** SNP associated with FPG, 2hPG, HbA1c, and BMI from the genome-wide association study after imputation analysis for prediabetes.

SNP	Chr (BP)	Gene	Minor/Majorallele	MAF	β (SE)	*p*-value	Potential function ^a^
2hPG associated							
rs13052524	21 (34075614)	MRPS6, SLC5A3	A/T	0.061	-0.909 (1.637)	4.35E-06	Promoter histone marks, Enhancer histone marks, DNAse, Motifs changed, Selected eQTL hits
rs62212118	21 (34093207)	MRPS6, SLC5A3	A/G	0.033	-0.927 (1.637)	4.03E-06	Enhancer histone marks, Motifs changed, Selected eQTL hits
HbA1C associated							
rs142013708	1 (30115880)	LINC01648, MATN1	C/A	0.030	-1.341 (1.547)	3.20E-06	/
rs140071694	1 (30116213)	LINC01648, MATN1	C/T	0.030	-1.341 (1.547)	3.20E-06	Motifs changed
rs150306839	1 (30116277)	LINC01648, MATN1	T/C	0.030	-1.341 (1.547)	3.20E-06	Motifs changed
rs138084074	1 (30116278)	LINC01648, MATN1	G/A	0.030	-1.341 (1.547)	3.20E-06	Motifs changed
rs142002616	1 (30116659)	LINC01648, MATN1	G/A	0.031	-1.357 (1.545)	1.60E-06	/
rs11853125	15 (91744926)	CRAT37, SLCO3A1	T/G	0.493	-1.619 (1.549)	4.78E-06	/
BMI associated							
rs11142842	9 (71478193)	TRPM3;TMEM2	G/A	0.306	-0.091 (1.465)	6.14E-07	Motifs changed
rs1891298	9 (71479613)	TRPM3;TMEM2	A/G	0.310	-0.086 (1.472)	4.33E-06	DNAse, Motifs changed
rs1891299	9 (71479750)	TRPM3;TMEM2	G/T	0.310	-0.087 (1.471)	3.92E-06	DNAse, Motifs changed
rs11142843	9 (71479866)	TRPM3;TMEM2	A/T	0.310	-0.087 (1.471)	3.92E-06	DNAse, Motifs changed
rs78432036	16 (83936738)	MLYCD;OSGIN1	T/C	0.060	0.067 (1.47)	3.00E-06	Enhancer histone marks, DNAse, Motifs changed

HbA1c, glycosylated hemoglobin; 2hPG, 2-hour postprandial glucose; BMI, body mass index. SNP, single nucleotide polymorphism; Chr, chromosome; BP, base pair; MAF, minor allele frequence; SE,standard error.

a Data from Haploreg (https://pubs.broadinstitute.org/mammals/haploreg/haploreg.php).

p < 5×10^-6^.

**Figure 2:**
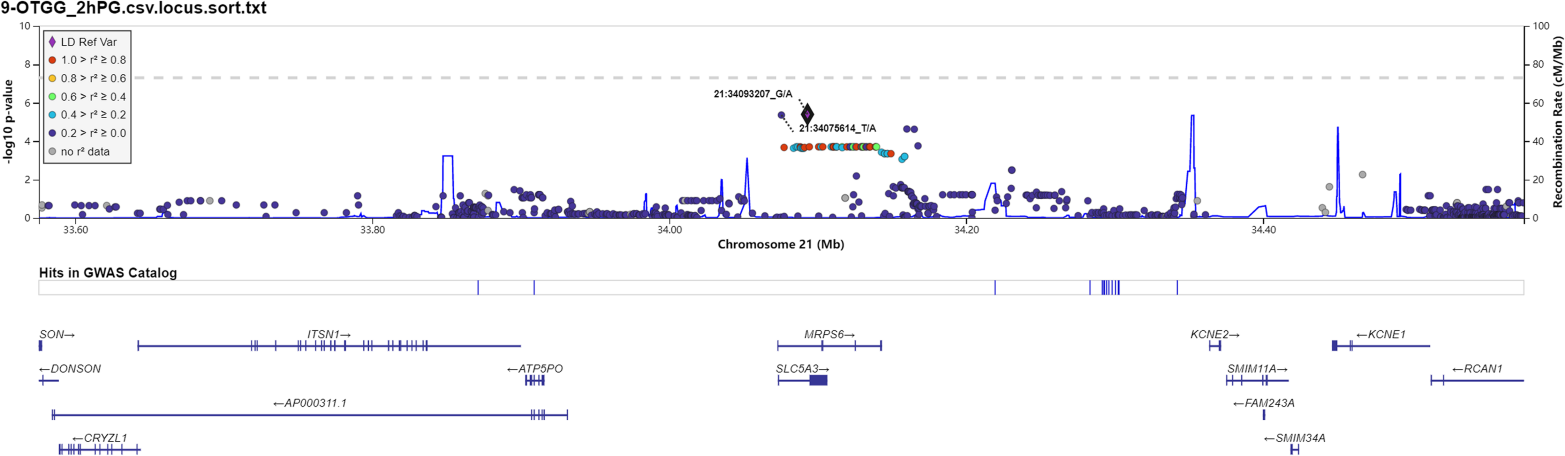
Regional association plots for newly identified loci associated with prediabetes after imputation with 2-h postprandial glucose (2hPG).

**Figure 3:**
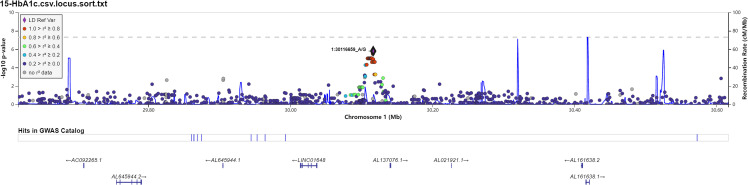
Data on the associated region on chromosome 1 to glycosylated hemoglobin (HbA1C).

**Figure 4:**
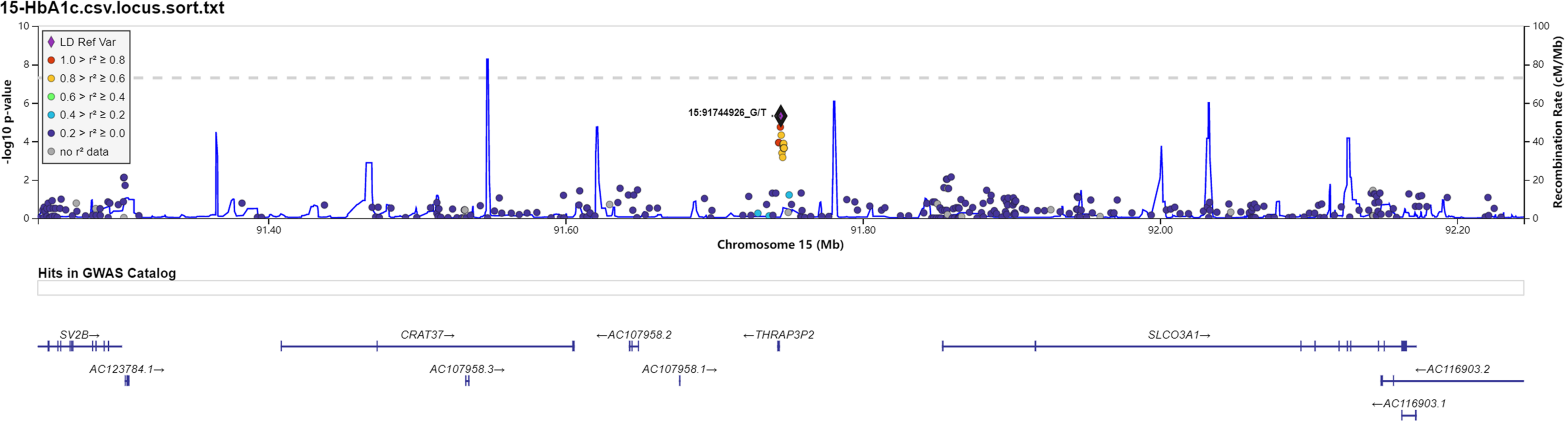
Data on the associated region on chromosome 15 include rs11853125 to glycosylated hemoglobin (HbA1C).

**Figure 5:**
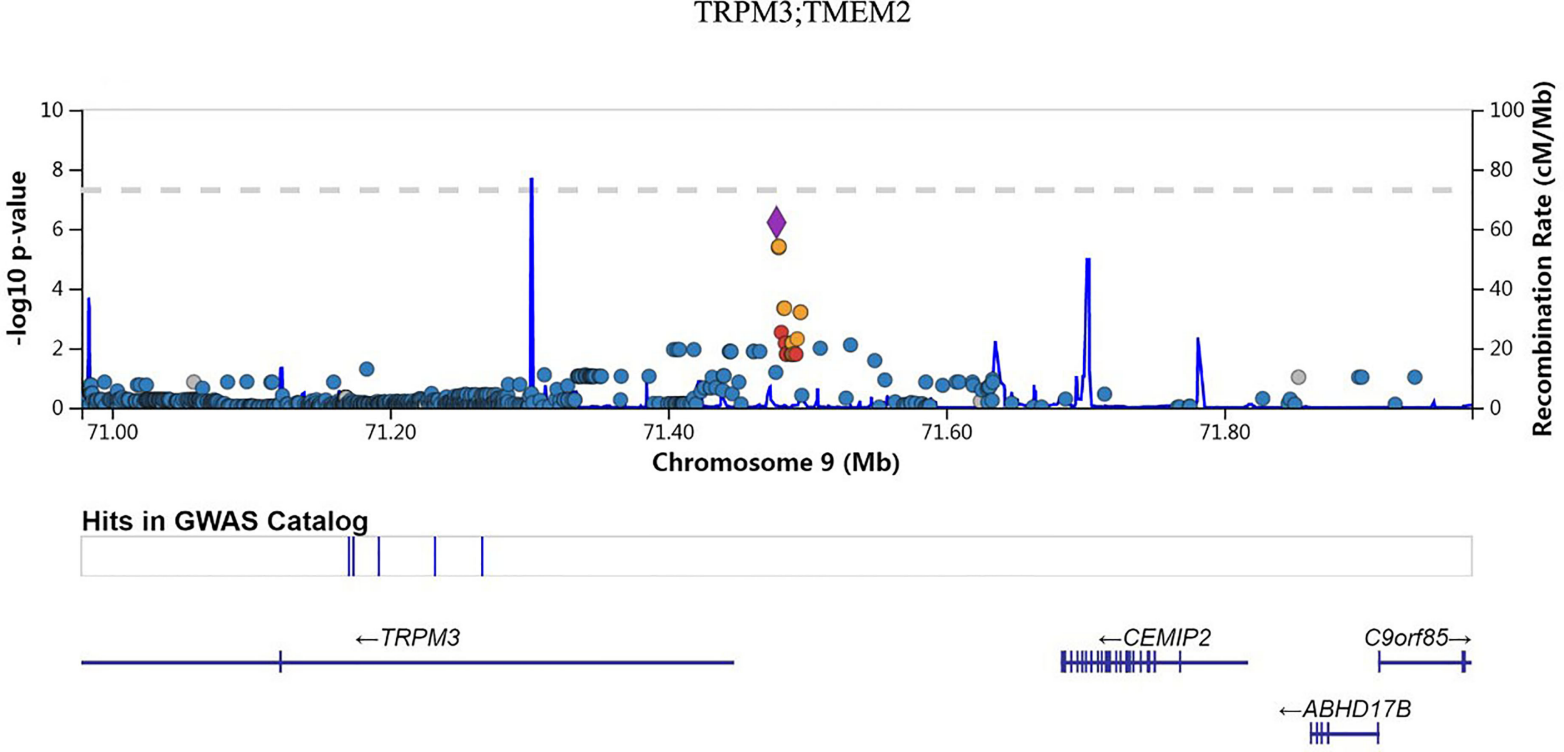
Data on the associated region on chromosome 9 include rs11142842 to BMI.

We also investigated the association of SNP with FPG, 2hPG, and HbA1C under the dominant model and recessive model; the results were presented in **Table S1**. In the dominant model, three SNPs (rs9550371, rs13052524, and rs62212118) were related to 2hPG (all *p* < 5 × 10^-6^), and 12 SNPs (rs142013708, rs140071694, rs150306839, rs138084074, rs142002616, rs1371810, rs1371809, rs4146607, rs4146606, rs13157326, rs6880621, and rs11745300) were associated with HbA1C (all *p* < 5 × 10^-6^). Besides, five SNPs (rs7624734, rs11142842, rs1891298, rs1891299, and rs11142843) were associated with BMI (all *p* < 5 × 10^-6^). The recessive model showed that 10 SNPs (rs4661250, rs3095307, rs3094203, rs3094202, rs3094201, rs3095302, rs3094200, rs3094198, rs3095301, and rs3131003) were associated with FPG (all *p* < 5 × 10^-6^), that 10 SNPs (rs201438706, rs12649862, rs41476645, rs12647991, rs12644845, rs12648073, rs12644936, rs12504012, rs34938732, and rs13119926) were related to 2hPG (all *p* < 5 × 10^-6^), and that two SNPs (rs946911 and rs2415427) were correlated with HbA1C (all *p* < 5 × 10^-6^). Moreover, two SNPs (rs62006357 and rs62008861) were correlated with BMI (all *p* < 5 × 10^-6^). We finally constructed the locus chart by LocalZoom online software, and the locus zoom plots of SNPs that were associated with 2hPG, HbA1C, and BMI are shown in **Figures 2**–**5**.

### Association Between the Genotype of Selected Single-Nucleotide Polymorphisms and Fasting Plasma Glucose, 2-h Postprandial Glucose, Glycosylated Hemoglobin, and Body Mass Index in Prediabetes

The association between the genotype of selected SNPs and FPG, 2hPG, HbA1C, and BMI in prediabetes was assessed, and the results were displayed in **Table 3**. The genotypes of rs13052524 and rs62212118 were associated with the level of 2hPG (*p* < 0.001). Besides, rs142013708, rs140071694, rs150306839, rs138084074, and rs142002616 were related to HbA1C levels (*p* = 0.004). Moreover, rs11142842, rs1891298, rs1891299, rs11142843, and rs78432036 were associated with BMI (*p* < 0.05).

**Table 3:** The genotype of candidate SNPs associated with FPG, 2hPG, and HbA1c, and BMI.

SNP	Genotype			*p*
2hPG associated				
rs13052524	AA	AT	TT	
	7.29 ± 1.44	7.58 ± 2.61	10.26 ± 4.45	<0.001
rs62212118	AG	GG		
	6.79 ± 1.92	10.22 ± 4.45		<0.001
HbA1c associated				
rs142013708	AA	AC		
	6.09 ± 1.35	5.29 ± 0.59		0.004
rs140071694	TT	CT		
	6.09 ± 1.36	5.29 ± 0.59		0.004
rs150306839	CT	CC		
	5.29 ± 0.59	6.09 ± 1.36		0.004
rs138084074	AA	AG		
	6.09 ± 1.36	5.29 ± 0.59		0.004
rs142002616	AA	AG		
	6.09 ± 1.36	5.29 ± 0.57		0.004
rs11853125	TT	GT	GG	
	6.31 ± 1.65	6.11 ± 1.31	5.85 ± 1.16	0.052
BMI associated				
rs11142842	AA	AG	GG	
	23.69 ± 3.9	24.76 ± 4.25	25.52 ± 3.16	0.009
rs1891298	AA	AG	GG	
	25.34 ± 3.3	24.75 ± 4.24	23.73 ± 3.89	0.017
rs1891299	TT	GT	GG	
	23.69 ± 3.9	24.75 ± 4.21	25.34 ± 3.3	0.013
rs11142843	AA	AT	TT	
	25.34 ± 3.3	24.75 ± 4.21	23.69 ± 3.9	0.013
rs78432036	TT	CT	CC	
	29.08 ± 0.96	26.05 ± 2.85	23.89 ± 4.21	0.001

HbA1c, glycosylated hemoglobin; 2hPG, 2 h postprandial glucose; BMI, body mass index. SNP, single nucleotide polymorphism.

p < 0.05.

## Discussion

T2D is a multifactorial disease affected by interactions of genetics, environment, and metabolism. The increases of FPG, 2hPG, and HbA1C levels are not only the symptoms of T2D but also independent risk factors for T2D (16, 17). The data of the epidemiological survey show that the risk of T2D begins even within the range of normal fasting blood glucose and increases exponentially in prediabetes (18–20). GWAS analysis is widely used in the association of genetic variants and phenotypes in T2D (21–23). Can genetic variants affect the phenotypes in prediabetes? In this study, we performed GWAS to determine the effect of genetic variants on FPG, 2hPG, HbA1C, and BMI in Hainan prediabetes. We found two susceptibility loci in *MRPS6*/*SLC5A3* genes associated with 2hPG, six loci in *LINC01648*, *MATN1*, *CRAT37*, and *SLCO3A1* genes associated with HbA1C, and five loci in *TRPM3/TMEM2* and *MLYCD/OSGIN1* correlated to BMI in Hainan prediabetes. To the best of our knowledge, our study is the first to detect the association of SNPs with FPG, 2hPG, HbA1C, and BMI in Hainan prediabetes.

Rs13052524 and rs62212118 are located in the intergenic region between Mitochondrial Ribosomal Protein S6 (*MRPS6*) and Solute Carrier Family 5 Member 3 (*SLC5A3*) at chromosome 21. *MRPS6* is a protein-coding gene. Previous GWASs have shown that *MRPS6* is associated with a variety of human diseases including diabetes (24, 25). *SLC5A3* is an inositol transporter that helps maintain osmotic balance in different tissues or organs, including kidney (26). An increasing number of studies indicate that *SLC5A3* plays an important role in diabetes-related metabolism (27, 28). Moreover, Beaney et al. (25) showed that *SLC5A3* polymorphisms were associated with human diseases such as coronary heart disease. Our study showed that rs13052524 and rs62212118 loci in the *MRPS6* gene were related to 2hPG level in the Hainan prediabetes, suggesting that these two loci might play a certain function in the regulation of 2hPG level. However, there are no related reports about rs13052524 and rs62212118 loci. HaploReg is a tool for exploring annotations of the non-coding genome at variants on haplotype blocks, such as candidate regulatory SNPs at disease-associated loci. Based on HaploReg, we found that rs13052524 and rs62212118 might be associated with enhancer histone marks, motifs changed, and selected eQTL hits, suggesting that the association of rs13052524 and rs62212118 with 2hPG level might be involved in regulating gene expression. However, the potential function of these polymorphisms needs to be confirmed by further experiments.

Rs142013708, rs140071694, rs150306839, rs138084074, and rs142002616 are located in the region between Long Intergenic Non-Protein-Coding RNA 1648 (*LINC01648*) and Matrilin-1 (*MATN1*) at chromosome 1. *MATN1* is a chondrocyte extracellular matrix protein, which serves as a marker of cell differentiation (29). Early studies have found that *MATN1* gene plays an important role in endochondral ossification (30, 31). Additionally, more and more studies have shown that *MANT1* genetic polymorphism is closely related to dental malocclusions of humans (32–34). In our study, we found that five novel loci in *MATN1* gene were related to HbA1C in prediabetes. Based on HaploReg, we found that rs140071694, rs150306839, and rs138084074 might be associated with changed motifs, suggesting that the association of rs140071694, rs150306839, and rs138084074 with HbA1C level might be involved in regulating changed motifs of gene. However, there are no reports on these loci, and the functions of these loci are still unknown that need to be further studied. The potential function of these polymorphisms needs to be confirmed by further experiments.

Rs11853125 is located at the region between Cervical Cancer-Associated Transcript 37 (*CRAT37*) and Solute Carrier Organic Anion Transporter Family Member 3A1 (*SLCO3A1*). *SLCO3A1* is an organic anion transporter that participates in the transport of non‐nucleoside reverse transcriptase inhibitor (NRTI) across the membrane (35, 36), which is associated with human diseases including intestinal perforation and primary hypertrophic osteoarthropathy. Furthermore, GWAS showed that the polymorphisms in this gene contributed to occurrence of some human diseases (37, 38). At present, there are no reports that this gene is related to diabetes. Here, we found that rs11853125 was related to HbA1C in prediabetes, suggesting that rs11853125 might contribute to the regulation of HbA1C level. However, the potential function needs to be explored.

Rs11142842, rs1891298, rs1891299, and rs11142843 are located in the region between transient receptor potential cation channel subfamily M member 3 (*TRPM3*) and transmembrane protein 2 (*TMEM2*) at chromosome 9q21.12-q21.13. TRPM3 belongs to the melastatin subfamily of TRP channels and represents a non-selective cation channel that can be activated by several different stimuli, including the neurosteroid pregnenolone sulfate, osmotic pressures, and heat (39). TRPM3 polymorphisms were reported to be associated with systemic sclerosis, aspirin-exacerbated respiratory disease (AERD), and developmental and epileptic encephalopathies (DEEs) (40–42). Transmembrane 2 (TMEM2) was a cell surface protein that possesses potent hyaluronidase activity (43). Here, we found that rs11142842, rs1891298, rs1891299, and rs11142843 were related to BMI in prediabetes. The potential function of these polymorphisms needs to be confirmed by further experiments.

Our study had some limitations. First, our sample size is relatively small in GWAS, and we would expand the sample size to verify our result in the future. Second, replication testing should be carried out to confirm the present data in the next work. Third, this study does not classify SNP with age/sex/BMI to investigate age/sex/BMI differences in SNP effects. Despite the above limitations, this is the first time to study the association between genetic polymorphism and the risk factors in Hainan Han Chinese prediabetes, and our study provides available information for further insight in the etiology of diabetes.

## Conclusions

In summary, we identified two novel SNPs (rs13052524 and rs62212118) in *MRPS6*/*SLC5A3* that were associated with 2hPG in Hainan prediabetes. Another six loci (rs142013708, rs140071694, rs150306839, rs138084074, rs142002616, and rs11853125) in four genes (*LINC01648*, *MATN1*, *CRAT37*, and *SLCO3A1*) were related to HbA1C.

## Data Availability Statement

The original contributions presented in the study are included in the article/**Supplementary Material**. Further inquiries can be directed to the corresponding author.

## Ethics Statement

The studies involving human participants were reviewed and approved by the Ethics Committee of Hainan Affiliated Hospital of Hainan Medical University [Med-Eth-Re (2019) 18]. The patients provided their written informed consent to participate in this study.

## Author Contributions

LHL conceived the study and wrote the article. TF, LL, and QO recruited and collected study samples. HZ and KC analyzed the data. All authors contributed to the article and approved the submitted version. HQ designed the study and revised the article.

## Funding

This study received the support of the major research and development program of Hainan Province (no. ZDYF2021SHFZ078), and received the support of project supported by Hainan Province Clinical Medical Center.

## Conflict of Interest

The authors declare that the research was conducted in the absence of any commercial or financial relationships that could be construed as a potential conflict of interest.

## Publisher’s Note

All claims expressed in this article are solely those of the authors and do not necessarily represent those of their affiliated organizations, or those of the publisher, the editors and the reviewers. Any product that may be evaluated in this article, or claim that may be made by its manufacturer, is not guaranteed or endorsed by the publisher.

## References

[1] Wild S Roglic G Green A Sicree R King H. Global Prevalence of Diabetes: Estimates for the Year 2000 and Projections for 2030. Diabetes Care (2004) 27(5):1047–53. doi: 10.2337/diacare.27.5.1047 15111519

[2] Sarwar N Gao P Seshasai SR Gobin R Kaptoge S Di Angelantonio E. Diabetes Mellitus, Fasting Blood Glucose Concentration, and Risk of Vascular Disease: A Collaborative Meta-Analysis of 102 Prospective Studies. Lancet (London England) (2010) 375(9733):2215–22. doi: 10.1016/S0140-6736(10)60484-9 PMC2904878 20609967

[3] Aguiree F Brown A Cho NH Dahlquist G Dodd S Dunning T. IDF Diabetes Atlas. sixth edition. International Diabetes Federation (2013). Available at: www.idf.org/diabetesatlas.

[4] Malchoff CD. Diagnosis and Classification of Diabetes Mellitus. Connecticut Med (2012) 35(Suppl 1):S64–71. doi: 10.2337/dc12-s064 PMC3632174 22187472

[5] Ohn JH Kwak SH Cho YM Lim S Jang HC Park KS. 10-Year Trajectory of β-Cell Function and Insulin Sensitivity in the Development of Type 2 Diabetes: A Community-Based Prospective Cohort Study. Lancet Diabetes Endocrinol (2016) 4(1):27–34. doi: 10.1016/S2213-8587(15)00336-8 26577716

[6] Thomas PPM Alshehri SM van Kranen HJ Ambrosino E. The Impact of Personalized Medicine of Type 2 Diabetes Mellitus in the Global Health Context. Personalized Med (2016) 13(4):381–93. doi: 10.2217/pme-2016-0029 29749811

[7] Choi JW Moon S Jang EJ Lee CH Park JS. Association of Prediabetes-Associated Single Nucleotide Polymorphisms With Microalbuminuria. PloS One (2017) 12(2):e0171367. doi: 10.1371/journal.pone.0171367 28158221 PMC5291388

[8] Soranzo N Sanna S Wheeler E Gieger C Radke D Dupuis J. Common Variants at 10 Genomic Loci Influence Hemoglobin A_1_(C) Levels *via* Glycemic and Nonglycemic Pathways. Diabetes (2010) 59(12):3229–39. doi: 10.2337/db10-0502 PMC2992787 20858683

[9] Ge S Wang Y Song M Li X Yu X Wang H. Type 2 Diabetes Mellitus: Integrative Analysis of Multiomics Data for Biomarker Discovery. Omics: J Integr Biol (2018) 22(7):514–23. doi: 10.1089/omi.2018.0053 30004843

[10] Ramos E Chen G Shriner D Doumatey A Gerry N Herbert A. Replication of Genome-Wide Association Studies (GWAS) Loci for Fasting Plasma Glucose in African-Americans. Diabetologia (2011) 54(4):783–8. doi: 10.1007/s00125-010-2002-7 PMC3052446 21188353

[11] Balkhiyarova Z Luciano R Kaakinen M Ulrich A Shmeliov A Bianchi M. Relationship Between Glucose Homeostasis and Obesity in Early Life-A Study of Italian Children and Adolescents. Hum Mol Genet (2021) ddab287. doi: 10.1093/hmg/ddab287 PMC8895752 34590674

[12] American Diabetes Association. Standards of medical care in diabetes--2010. Diabetes Care (2010) 33:(Suppl 1):S11–61. doi: 10.2337/dc10- PMC2797382 20042772

[13] Hurskainen AR Virtanen JK Tuomainen TP Nurmi T Voutilainen S. Association of Serum 25-Hydroxyvitamin D With Type 2 Diabetes and Markers of Insulin Resistance in a General Older Population in Finland. Diabetes/Metabol Res Rev (2012) 28(5):418–23. doi: 10.1002/dmrr.2286 22318870

[14] Marchini J Howie B Myers S McVean G Donnelly P. A New Multipoint Method for Genome-Wide Association Studies by Imputation of Genotypes. Nat Genet (2007) 39(7):906–13. doi: 10.1038/ng2088 17572673

[15] Das S Forer L Schönherr S Sidore C Locke AE Kwong A. Next-Generation Genotype Imputation Service and Methods. Nat Genet (2016) 48(10):1284–7. doi: 10.1038/ng.3656 PMC5157836 27571263

[16] Mozaffary A Asgari S Tohidi M Kazempour-Ardebili S Azizi F Hadaegh F. Change in Fasting Plasma Glucose and Incident Type 2 Diabetes Mellitus: Results From a Prospective Cohort Study. BMJ Open (2016) 6(5):e010889. doi: 10.1136/bmjopen-2015-010889 PMC4885425 27217283

[17] Zaura E Keijser BJ Huse SM Crielaard W. Defining the Healthy “Core Microbiome” of Oral Microbial Communities. BMC Microbiol (2009) 9(1):259. doi: 10.1186/1471-2180-9-259 20003481 PMC2805672

[18] Mason CC Hanson RL Knowler WC. Progression to Type 2 Diabetes Characterized by Moderate Then Rapid Glucose Increases. Diabetes (2007) 56(8):2054–61. doi: 10.2337/db07-0053 17473220

[19] Choi SH Kim TH Lim S Park KS Jang HC Cho NH. Hemoglobin A1c as a Diagnostic Tool for Diabetes Screening and New-Onset Diabetes Prediction: A 6-Year Community-Based Prospective Study. Diabetes Care (2011) 34(4):944–9. doi: 10.2337/dc10-0644 PMC3064055 21335372

[20] Tabák AG Herder C Rathmann W Brunner EJ Kivimäki M. Prediabetes: A High-Risk State for Diabetes Development. Lancet (London England) (2012) 379(9833):2279–90. doi: 10.1016/S0140-6736(12)60283-9 PMC3891203 22683128

[21] Liu C Wu Y Li H Qi Q Langenberg C Loos RJ. MTNR1B Rs10830963 is Associated With Fasting Plasma Glucose, HbA1C and Impaired Beta-Cell Function in Chinese Hans From Shanghai. BMC Med Genet (2010) 11:59. doi: 10.1186/1471-2350-11-59 20398260 PMC2873324

[22] Franklin CS Aulchenko YS Huffman JE Vitart V Hayward C Polašek O. The TCF7L2 Diabetes Risk Variant is Associated With HbA_1_(C) Levels: A Genome-Wide Association Meta-Analysis. Ann Hum Genet (2010) 74(6):471–8. doi: 10.1111/j.1469-1809.2010.00607.x 20849430

[23] Li H Gan W Lu L Dong X Han X Hu C. A Genome-Wide Association Study Identifies GRK5 and RASGRP1 as Type 2 Diabetes Loci in Chinese Hans. Diabetes (2013) 62(1):291–8. doi: 10.2337/db12-0454 PMC3526061 22961080

[24] Szpakowicz A Kiliszek M Pepiński W Waszkiewicz E Franaszczyk M Skawrońska M. The Rs9982601 Polymorphism of the Region Between the SLC5A3/MRPS6 and KCNE2 Genes Associated With a Prevalence of Myocardial Infarction and Subsequent Long-Term Mortality. Polskie Archiwum Medycyny Wewnetrznej (2015) 125(4):240–8. doi: 10.20452/pamw.2780 25697262

[25] Beaney KE Smith AJP Folkersen L Palmen J Wannamethee SG Jefferis BJ. Functional Analysis of the Coronary Heart Disease Risk Locus on Chromosome 21q22. Dis Markers (2017) 2017:1096916. doi: 10.1155/2017/1096916 28458444 PMC5387827

[26] Schneider S. Inositol Transport Proteins. FEBS Lett (2015) 589(10):1049–58. doi: 10.1016/j.febslet.2015.03.012 25819438

[27] Li SYT Cheng STW Zhang D Leung PS. Identification and Functional Implications of Sodium/Myo-Inositol Cotransporter 1 in Pancreatic β-Cells and Type 2 Diabetes. Diabetes (2017) 66(5):1258–71. doi: 10.2337/db16-0880 28202581

[28] Van Steenbergen A Balteau M Ginion A Ferté L Battault S Ravenstein CM. Sodium-Myoinositol Cotransporter-1, SMIT1, Mediates the Production of Reactive Oxygen Species Induced by Hyperglycemia in the Heart. Sci Rep (2017) 7:41166. doi: 10.1038/srep41166 28128227 PMC5269587

[29] Deák F Wagener R Kiss I Paulsson M. The Matrilins: A Novel Family of Oligomeric Extracellular Matrix Proteins. Matrix Biol (1999) 18(1):55–64. doi: 10.1016/S0945-053X(98)00006-7 10367731

[30] Klatt A Paulsson M Wagener R. Expression of Matrilins During Maturation of Mouse Skeletal Tissues. Matrix Biol (2002) 21(3):289–96. doi: 10.1016/S0945-053X(02)00006-9 12009334

[31] Hyde G Dover S Aszodi A Wallis G Boot-Handford R. Lineage Tracing Using Matrilin-1 Gene Expression Reveals That Articular Chondrocytes Exist as the Joint Interzone Forms. Dev Biol (2007) 304(2):825–33. doi: 10.1016/j.ydbio.2007.01.026 PMC2795868 17313942

[32] Balkhande PB Lakkakula B Chitharanjan AB. Relationship Between Matrilin-1 Gene Polymorphisms and Mandibular Retrognathism. Am J Orthodontics Dentofacial Orthopedics (2018) 153(2):255–61.e1. doi: 10.1016/j.ajodo.2017.06.023 29407503

[33] Yilmaz AD Yazicioğlu D Tuzuner Oncul MA Ereş G Sayan NB. Association of Matrilin-3 Gene Polymorphism With Temporomandibular Joint Internal Derangement. Genet Testing Mol Biomarkers (2016) 20(10):563–8. doi: 10.1089/gtmb.2016.0037 27533128

[34] Jang JY Park EK Ryoo HM Shin HI Kim TH Jang JS. Polymorphisms in the Matrilin-1 Gene and Risk of Mandibular Prognathism in Koreans. J Dental Res (2010) 89(11):1203–7. doi: 10.1177/0022034510375962 20739701

[35] Janneh O Anwar T Jungbauer C Kopp S Khoo SH Back DJ. P-Glycoprotein, Multidrug Resistance-Associated Proteins and Human Organic Anion Transporting Polypeptide Influence the Intracellular Accumulation of Atazanavir. Antiviral Ther (2009) 14(7):965–74. doi: 10.3851/IMP1399 19918100

[36] Purcet S Minuesa G Molina-Arcas M Erkizia I Casado FJ Clotet B. 3’-Azido-2’,3’-Dideoxythymidine (Zidovudine) Uptake Mechanisms in T Lymphocytes. Antiviral Ther (2006) 11(6):803–11. 17310825

[37] Coelho AV Silva SP de Alencar LC Stocco G Crovella S Brandão LA. ABCB1 and ABCC1 Variants Associated With Virological Failure of First-Line Protease Inhibitors Antiretroviral Regimens in Northeast Brazil Patients. J Clin Pharmacol (2013) 53(12):1286–93. doi: 10.1002/jcph.165 23996099

[38] Klein KM Bromhead CJ Smith KR O’Callaghan CJ Corcoran SJ Heron SE. Autosomal Dominant Vasovagal Syncope: Clinical Features and Linkage to Chromosome 15q26. Neurology (2013) 80(16):1485–93. doi: 10.1212/WNL.0b013e31828cfad0 23589636

[39] Held K Tóth BI. TRPM3 in Brain (Patho)Physiology. Front Cell Dev Biol (2021) 9:635659. doi: 10.3389/fcell.2021.635659 33732703 PMC7959729

[40] Oztuzcu S Onat AM Pehlivan Y Alibaz-Oner F Donmez S Cetin GY. Association of TRPM Channel Gene Polymorphisms With Systemic Sclerosis. In Vivo (Athens Greece) (2015) 29(6):763–70. 26546534

[41] Narayanankutty A Palma-Lara I Pavón-Romero G Pérez-Rubio G Camarena Á Teran LM. Association of TRPM3 Polymorphism (Rs10780946) and Aspirin-Exacerbated Respiratory Disease (AERD). Lung (2016) 194(2):273–9. doi: 10.1007/s00408-016-9852-9 26891941

[42] Kang Q Yang L Liao H Yang S Kuang X Ning Z. A Chinese Patient With Developmental and Epileptic Encephalopathies (DEE) Carrying a TRPM3 Gene Mutation: A Paediatric Case Report. BMC Pediatr (2021) 21(1):256. doi: 10.1186/s12887-021-02719-8 34074259 PMC8167971

[43] Yamaguchi Y Yamamoto H Tobisawa Y Irie F. TMEM2: A Missing Link in Hyaluronan Catabolism Identified? Matrix Biol (2019) 78-79:139–46. doi: 10.1016/j.matbio.2018.03.020 PMC6314907 29601864

