# Supplementary figures and images for: Genetic Variants Relate to Fasting Plasma Glucose, 2-Hour Postprandial Glucose, Glycosylated Hemoglobin, and BMI in Prediabetes
Source: Front Endocrinol (Lausanne). 2022 Mar 1;13:778069. doi: 10.3389/fendo.2022.778069 (PMC8923657; doi:10.3389/fendo.2022.778069)

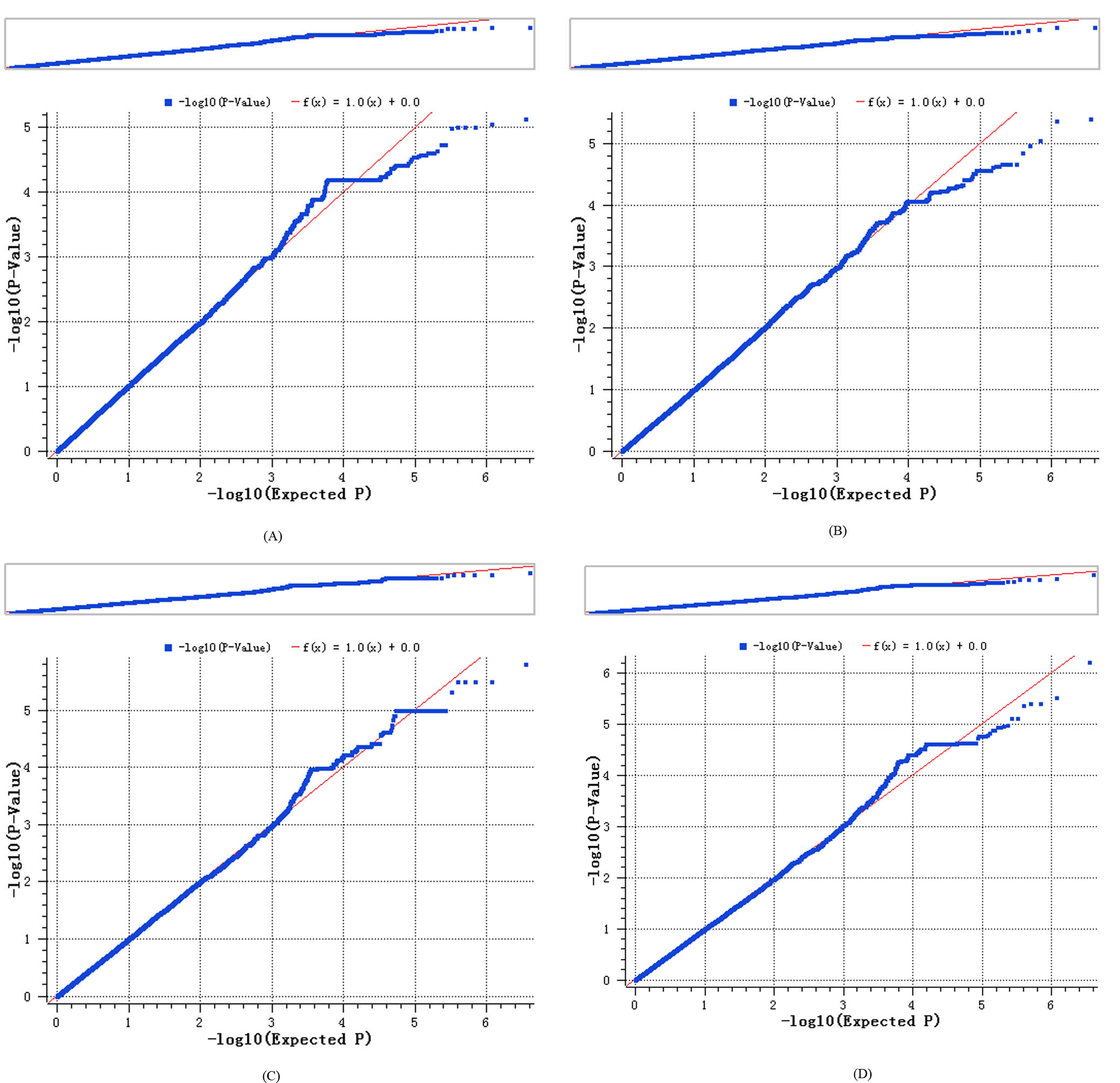

Supplement: Supplementary file 1 [file Image_1.jpeg]
