# Supplementary material for: Genetic Variants Relate to Fasting Plasma Glucose, 2-Hour Postprandial Glucose, Glycosylated Hemoglobin, and BMI in Prediabetes
Source: Front Endocrinol (Lausanne). 2022 Mar 1;13:778069. doi: 10.3389/fendo.2022.778069 (PMC8923657; doi:10.3389/fendo.2022.778069)
Supplement: Supplementary file 2 [file Table_1.pdf]

Table S1 SNP associated with FPG, 2hPG, HbA1c and BMI from the genome-wide association study after imputation analysis for prediabetes

| SNP | Chr (BP) | Gene | Minor/Major allele | MAF | β (SE) | *p* Value |
|---|---|---|---|---|---|---|
| Dominant model | | | | | | |
| 2hPG associated | | | | | | |
| rs9550371 | 13 (30690780) | USPL1, ALOX5AP | G/A | 0.167 | -1.149 (1.637) | 3.88E-06 |
| rs13052524 | 21 (34075614) | MRPS6, SLC5A3 | A/T | 0.061 | -0.914 (1.635) | 2.09E-06 |
| rs62212118 | 21 (34093207) | MRPS6, SLC5A3 | A/G | 0.033 | -0.927 (1.637) | 4.03E-06 |
| HbA1c associated | | | | | | |
| rs142013708 | 1 (30115880) | LINC01648, MATN1 | C/A | 0.030 | -1.341 (1.547) | 3.20E-06 |
| rs140071694 | 1 (30116213) | LINC01648, MATN1 | C/T | 0.030 | -1.341 (1.547) | 3.20E-06 |
| rs150306839 | 1 (30116277) | LINC01648, MATN1 | T/C | 0.030 | -1.341 (1.547) | 3.20E-06 |
| rs138084074 | 1 (30116278) | LINC01648, MATN1 | G/A | 0.030 | -1.341 (1.547) | 3.20E-06 |
| rs142002616 | 1 (30116659) | LINC01648, MATN1 | G/A | 0.031 | -1.357 (1.545) | 1.60E-06 |
| rs1371810 | 5 (34503247) | NONE, RAI14 | T/C | 0.347 | -1.172 (1.547) | 2.70E-06 |
| rs1371809 | 5 (34503441) | NONE, RAI14 | G/T | 0.349 | -1.156 (1.547) | 2.59E-06 |
| rs4146607 | 5 (34503676) | NONE, RAI14 | C/A | 0.349 | -1.156 (1.547) | 2.59E-06 |
| rs4146606 | 5 (34503902) | NONE, RAI14 | C/T | 0.349 | -1.156 (1.547) | 2.59E-06 |
| rs13157326 | 5 (34504172) | NONE, RAI14 | A/G | 0.349 | -1.156 (1.547) | 2.59E-06 |
| rs6880621 | 5 (34504277) | NONE, RAI14 | G/A | 0.349 | -1.162 (1.547) | 2.49E-06 |
| rs11745300 | 5 (34504563) | NONE, RAI14 | G/C | 0.349 | -1.156 (1.547) | 2.59E-06 |
| BMI associated | | | | | | |
| rs7624734 | 3 (39067012) | WDR48 | A/G | 0.340 | -0.184 (1.471) | 2.58E-06 |
| rs11142842 | 9 (71478193) | TRPM3;TMEM2 | G/A | 0.306 | -0.121 (1.465) | 5.31E-07 |
| rs1891298 | 9 (71479613) | TRPM3;TMEM2 | A/G | 0.310 | -0.124 (1.469) | 2.13E-06 |
| rs1891299 | 9 (71479750) | TRPM3;TMEM2 | G/T | 0.310 | -0.125 (1.469) | 1.96E-06 |
| rs11142843 | 9 (71479866) | TRPM3;TMEM2 | A/T | 0.310 | -0.125 (1.469) | 1.96E-06 |
| Recessive model | | | | | | |

| | | | | | | |
|---|---|---|---|---|---|---|
| FPG associated | | | | | | |
| rs4661250 | 1 (223225986) | SUSD4 | G/A | 0.204 | -1.243 (0.843) | 4.64E-06 |
| rs3095307 | 6 (31124272) | PSORS1C1 | G/C | 0.351 | -1.304 (0.842) | 2.92E-06 |
| rs3094203 | 6 (31124764) | PSORS1C1 | A/G | 0.351 | -1.304 (0.842) | 2.92E-06 |
| rs3094202 | 6 (31124770) | PSORS1C1 | A/G | 0.338 | -1.313 (0.842) | 4.31E-06 |
| rs3094201 | 6 (31124986) | PSORS1C1 | A/G | 0.352 | -1.304 (0.842) | 2.92E-06 |
| rs3095302 | 6 (31125289) | PSORS1C1 | G/A | 0.352 | -1.304 (0.842) | 2.92E-06 |
| rs3094200 | 6 (31125355) | PSORS1C1 | A/G | 0.352 | -1.304 (0.842) | 2.92E-06 |
| rs3094198 | 6 (31125550) | PSORS1C1 | A/G | 0.352 | -1.304 (0.842) | 2.92E-06 |
| rs3095301 | 6 (31125579) | PSORS1C1 | T/C | 0.338 | -1.313 (0.842) | 4.31E-06 |
| rs3131003 | 6 (31125705) | PSORS1C1 | G/A | 0.336 | -1.313 (0.842) | 4.31E-06 |
| 2hPG associated | | | | | | |
| rs201438706 | 4 (77421653) | CCNG2, CXCL13 | T/A | 0.301 | -0.906 (1.635) | 2.66E-06 |
| rs12649862 | 4 (77422666) | CCNG2, CXCL13 | A/G | 0.301 | -0.906 (1.635) | 2.66E-06 |
| rs41476645 | 4 (77423231) | CCNG2, CXCL13 | T/G | 0.301 | -0.906 (1.635) | 2.66E-06 |
| rs12647991 | 4 (77423575) | CCNG2, CXCL13 | C/A | 0.301 | -0.906 (1.635) | 2.66E-06 |
| rs12644845 | 4 (77423590) | CCNG2, CXCL13 | T/C | 0.301 | -0.906 (1.635) | 2.66E-06 |
| rs12648073 | 4 (77423855) | CCNG2, CXCL13 | G/A | 0.301 | -0.906 (1.635) | 2.66E-06 |
| rs12644936 | 4 (77423966) | CCNG2, CXCL13 | T/C | 0.301 | -0.906 (1.635) | 2.66E-06 |
| rs12504012 | 4 (77424479) | CCNG2, CXCL13 | G/A | 0.300 | -0.906 (1.635) | 2.66E-06 |
| rs34938732 | 4 (77424709) | CCNG2, CXCL13 | A/G | 0.302 | -0.895 (1.634) | 1.61E-06 |
| rs13119926 | 4 (77425298) | CCNG2, CXCL13 | A/G | 0.302 | -0.895 (1.634) | 1.61E-06 |
| HbA1c associated | | | | | | |
| rs946911 | 14 (38039549) | LINC00517, SSTR1 | C/T | 0.200 | -1.466 (1.544) | 1.26E-06 |
| rs2415427 | 14 (38040029) | LINC00517, SSTR1 | G/A | 0.200 | -1.466 (1.544) | 1.26E-06 |
| BMI associated | | | | | | |
| rs62006357 | 15 (80252110) | LINC01314 | G/A | 0.453 | 0.074 (1.471) | 4.36E-06 |
| rs62008861 | 15 (80253711) | LINC01314;LINC00927 | G/A | 0.451 | 0.085 (1.47) | 3.39E-06 |

FPG: fasting plasma glucose, HbA1c: glycosylated hemoglobin, 2hPG: 2-hour postprandial glucose, BMI: body mass index.
$p < 5\times10^{-6}$
